# Rag1 Deficiency Impairs Arteriogenesis in Mice

**DOI:** 10.3390/ijms241612839

**Published:** 2023-08-16

**Authors:** Konda Kumaraswami, Christoph Arnholdt, Elisabeth Deindl, Manuel Lasch

**Affiliations:** 1 Walter-Brendel-Centre of Experimental Medicine, University Hospital, Ludwig-Maximilians-Universität München, 81377 Munich, Germany; kumaraswami.konda@med.uni-muenchen.de (K.K.); christoph.arnholdt@med.uni-muenchen.de (C.A.); manuel.lasch@med.uni-muenchen.de (M.L.); 2 Medical Clinic I, Department of Cardiology, University Hospital, Ludwig Maximilian University, 81377 Munich, Germany; 3 Biomedical Center, Institute of Cardiovascular Physiology and Pathophysiology, Ludwig-Maximilians-Universität München, 82152 Planegg-Martinsried, Germany; 4 Department of Otorhinolaryngology, Head and Neck Surgery, University Hospital, Ludwig Maximilian University, 81377 Munich, Germany

**Keywords:** Rag1, absence of T cells, absence of B cells, arteriogenesis, macrophage polarization

## Abstract

Increasing evidence suggests that lymphocytes play distinct roles in inflammation-induced tissue remodeling and tissue damage. Arteriogenesis describes the growth of natural bypasses from pre-existing collateral arteries. This process compensates for the loss of artery function in occlusive arterial diseases. The role of innate immune cells is widely understood in the process of arteriogenesis, whereas the role of lymphocytes remains unclear and is the subject of the present study. To analyze the role of lymphocytes, we induced arteriogenesis in recombination activating gene-1 (Rag1) knockout (KO) mice by unilateral ligation of the femoral artery. The lack of functional lymphocytes in Rag1 KO mice resulted in reduced perfusion recovery as shown by laser Doppler imaging. Additionally, immunofluorescence staining revealed a reduced vascular cell proliferation along with a smaller inner luminal diameter in Rag1 KO mice. The perivascular macrophage polarization around the growing collateral arteries was shifted to more pro-inflammatory M1-like polarized macrophages. Together, these data suggest that lymphocytes are crucial for arteriogenesis by modulating perivascular macrophage polarization.

## 1. Introduction

The leading causes of death worldwide are vascular occlusive diseases, including myocardial infarction, stroke, and peripheral artery diseases [1]. The existing options to treat these vascular occlusive diseases are percutaneous transluminal angioplasty or bypass surgery [2]. Despite medical advances, a significant number of patients suffer from failed surgery or post-surgery complications. Interestingly, the human body is able to grow pre-existing collateral arteries into fully functional arteries to compensate for the loss of artery function caused by occlusion or stenosis [2]. This growth of natural bypasses is called arteriogenesis [3]. When normal blood flow through a major artery is impaired arteriogenesis is crucial for preserving organ function. Despite being the only natural way to compensate for the functional loss of an artery, the development of collateral arteries is a time-consuming process [3]. It is necessary to fully comprehend the intricate mechanism of arteriogenesis in order to develop potential non-invasive alternative therapeutic approaches to promote vessel growth [3].

Occlusion of an artery increases fluid shear stress in pre-existing collaterals presenting the primary physiological stimulus for endothelium activation and arteriogenesis [4]. To dissect the molecular mechanisms involved in arteriogenesis, it is crucial to understand the mechanosensory complex signal transduction. In the case of increased shear stress, previous results identified extracellular RNA (eRNA) (released from endothelial cells) as a messenger. This eRNA initiates the inflammatory process by activating the vascular endothelial growth factor receptor 2 (VEGFR2). Such activation results in the release of von Willebrand Factor (vWF) from Weibel–Palade bodies. vWF in turn stimulates the platelet receptor GPIbα, leading to platelet activation and platelet–neutrophil aggregate (PNA) formation [4]. Upon activation and extravasation, neutrophils activate mast cells via reactive oxygen species. These activated mast cells are required for recruiting leukocytes, especially macrophages, to the perivascular space. This recruitment increases the bioavailability of growth-promoting factors, enhancing the process of arteriogenesis [5].

Whereas the role of cells of the innate immune system is well-described [6], little is known about the function of adaptive immune cells for the process of arteriogenesis. B- and T cells are components of the adaptive immune system that have various functions [6]. T cells are categorized into two major subtypes: CD4^+^ and CD8^+^, which are responsible for immune activation and cytotoxic functions, respectively. Regulatory T cells (Treg), a subtype, are important for immune tolerance and maintaining homeostasis [7]. B cells are well known for their ability to secrete antibodies as part of the humoral immune response. However, they have been reported to have antibody-independent functions in sterile inflammation [8,9].

A protective role of Foxp3^+^ Treg has been demonstrated in a mouse model of myocardial infarction [9]. Using Foxp3 DTR ablation and Treg depletion, the authors found aggravated tissue remodeling associated with M1-like macrophage polarization. Conversely, Treg cell activation restored M2-like macrophage polarization and cardiac remodeling [10]. The recruitment of perivascular macrophages is particularly important throughout this phase [10].

A close relation between innate and adaptive immune cells during remodeling processes is already established. B cells, for example, chemo-attract neutrophils and induce inflammation and fibrosis [11]. However, IL-10^+^ regulatory B cells show anti-inflammatory properties and facilitate cardiac remodeling, and IL-10–producing B cells are enriched in murine pericardial adipose tissues and ameliorate the outcome of acute myocardial infarction [12]. These results indicate that functional differences in lymphocyte subsets are crucial in determining the remodeling outcome [13].

Based on current knowledge, it is evident that the adaptive immune system is involved in cardiovascular diseases; however, its role during the process of arteriogenesis is barely understood. While data on B cell function are lacking, the role of T cells in arteriogenesis is discussed with contradictory findings [14,15]. However, none of the studies have examined vascular proliferation and macrophage polarization, both of which are essential for arteriogenesis. 

In the present study, we investigated the effect of the absence of B cells and T cells in Rag1 KO mice by assessing perfusion recovery, vascular cell proliferation, and luminal diameter. Furthermore, we investigated the impact of Rag1 deficiency on perivascular macrophage accumulation and polarization, which are pivotal for effective arteriogenesis.

## 2. Results

### 2.1. Impaired Perfusion Recovery and Arterial Growth in Rag1 KO Mice

**Figure 1:**
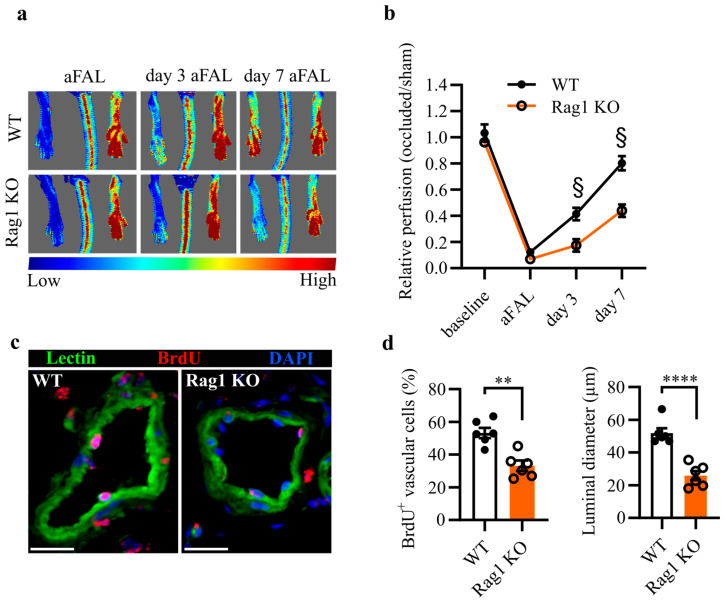
Impaired arteriogenesis in Rag1 deficient mice. (**a**) Representative laser Doppler flux images of WT and Rag1 KO mice immediately after femoral artery ligation (aFAL) (**left**), at day 3 (**middle**), and day 7 (**right**). The flux scale indicates an increase in perfusion from blue (low) to red (high). (**b**) Relative perfusion of WT and Rag1 KO mice at baseline, aFAL, at day 3 and day 7 aFAL. n = 6; data represent means ± S.E.M.; § = *p* < 0.0001 by two-way ANOVA multiple comparison test with Bonferroni correction. (**c**) Epifluorescence images of WT and Rag1 KO mice adductor muscles at day 7 aFAL stained for BrdU, lectin, and DAPI (nuclei). Proliferating vascular cells were identified as BrdU^+^ nucleated cells residing on the vascular wall. Scale bar: 20 μm. (**d**) Bar graphs showing the number of BrdU^+^ vascular cells (**left**) and luminal diameter of collateral arteries (**right**). Each dot represents the mean data of 6–10 collateral artery sections per mouse. n = 6 mice; data are means ± S.E.M.; ** *p* < 0.01, **** *p* < 0.0001 analyzed by unpaired Student’s *t*-test.

Innate immune cells are required to ensure effective arteriogenesis [16]. To explore whether acquired immune cells may also play a role in this process, we used Rag1 KO mice, a well-established model that lacks both mature B- and T lymphocytes [17]. Arteriogenesis was induced in both WT mice and Rag1 KO mice by FAL. We analyzed perfusion recovery before FAL (baseline), immediately aFAL, and on day 3 and day 7 aFAL. We observed a significant reduction in perfusion recovery in Rag1 KO mice in comparison to WT mice on both day 3 and day 7 aFAL (Figure 1a,b).

To verify whether the decreased perfusion recovery was due to insufficient vascular cell proliferation and vascular growth, WT mice and Rag1 KO mice were treated with the proliferative marker, BrdU. Vascular cell proliferation and luminal diameter were evaluated through immunofluorescence staining for BrdU and lectin in adductor muscles collected on day 7 aFAL (Figure 1c). In accordance with our perfusion recovery data, Rag1 KO mice showed a significantly decreased vascular cell proliferation and a smaller inner luminal diameter compared to WT mice (Figure 1c,d).

### 2.2. Altered Perivascular Macrophage Polarization in Rag1 KO Mice

**Figure 2:**
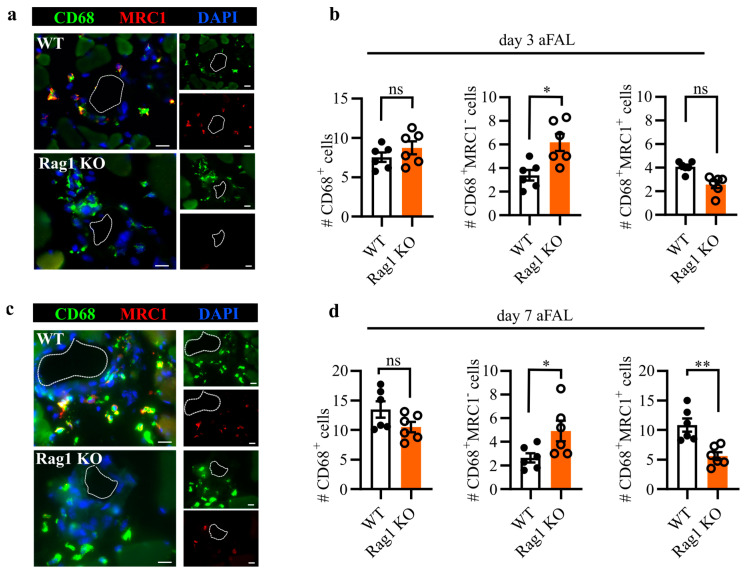
Perivascular macrophage polarization in Rag1 KO mice. Representative immunofluorescence images of macrophages in the perivascular space of growing collateral arteries in WT and Rag1 KO mice at day 3 (**a**) and day 7 (**c**) after femoral artery ligation (aFAL). The arterial lumen is indicated by a dashed line. Scale bar: 20 μm. Bar graphs depicting the number of CD68^+^ macrophages, CD68^+^MRC1^−^ (M1-like-polarized), and CD68^+^MRC1^+^ (M2-like-polarized) macrophages at day 3 (**b**) and day 7 aFAL (**d**). The *y*-axis in (**b**,**d**) depicts the absolute number of perivascular macrophages per collateral artery. Data are means ± S.E.M. Each dot represents the mean of 6–10 collateral artery sections per mouse. * *p* < 0.05, ** *p* < 0.01, ns (no significance) determined by unpaired Student’s *t*-test, n = 6 mice per group.

Macrophages are known to act as key regulators of inflammation and tissue regeneration [18]. Studies focusing on the spatial and temporal distribution of perivascular macrophages have revealed their critical role in arteriogenesis [19]. To understand the perivascular macrophage dynamics in Rag1 deficient mice, we investigated the number and polarization of macrophages in the perivascular space of growing collateral arteries at day 3 (Figure 2a,b) and day 7 aFAL (Figure 2c,d). We used CD68 as a pan-macrophage marker while the MRC1 marker was used to distinguish between inflammatory (M1-like) and regenerative (M2-like) macrophage polarization. Accordingly, CD68^+^MRC1^−^ macrophages were defined as M1-like polarized, while CD68^+^MRC1^+^ were defined as M2-like polarized macrophages. Our immunofluorescence analysis revealed that the number of perivascular macrophages remained statistically similar between Rag1 KO and WT mice across both evaluated time points. However, macrophage polarization analysis revealed that Rag1 KO mice showed a significantly increased number of macrophages with M1-like polarization at day 3 and day 7 aFAL (Figure 2b,d). The number of M2-like polarized macrophages showed a decreasing trend on day 3 aFAL in Rag KO mice (Figure 2a,b); however, the value was significant at day 7 aFAL (Figure 2c,d).

## 3. Discussion

The contribution of the adaptive immune system in arteriogenesis is not fully understood. In this study, using the Rag1 KO mice, we investigated arteriogenesis using perfusion recovery, vascular proliferation, and perivascular macrophage polarization. Our data suggest that the adaptive immune system plays an important role in arteriogenesis by modulating macrophage polarization which in turn is essential for vascular cell proliferation.

Arteriogenesis is an adaptive response to arterial vessel occlusion or stenosis protecting organs from ischemic damage [3]. This process is triggered by fluid shear stress and leukocyte-supplied growth-promoting factors [3,4,16]. Among leukocytes, neutrophils and macrophages are reported to be essential for arterial remodeling [16]. Therefore, the majority of investigations have focused on innate immune cells. The Rag1 KO model provides a mouse model to investigate the functional relevance of lymphocytes [17]. A study by Zouggari et al. reported the use of Rag1 KO mice as an adoptive cell transfusion model in the context of arteriogenesis [14]. However, this study did not provide data on vascular proliferation and macrophage polarization, which are crucial for arteriogenesis. 

The aim of this study was to conduct a thorough analysis of arteriogenesis in Rag1 KO mice. We investigated perfusion recovery, vascular cell proliferation, and vascular growth. The Rag1 KO mice showed decreased arteriogenesis compared to WT mice, demonstrated by the reduced perfusion recovery, decreased vascular cell proliferation, and reduced luminal diameter, suggesting that lymphocytes play an important role in arteriogenesis. 

As a complex process, arteriogenesis can be divided into an early inflammatory phase followed by a regenerative phase. Two predominant macrophage phenotypes have been implicated in arteriogenesis [19]: firstly, M1-like polarized macrophages are crucial during the inflammatory phase and can be identified by the absence of surface marker MRC1 [20]. Secondly, M2-like polarized regenerative macrophages, which are identifiable through the presence of MRC1 [21,22]. Analyzing spatial and temporal macrophage dynamics revealed that the magnitude of arteriogenesis is directly proportional to the optimal balance of M1-like and M2-like macrophages [23].

Rag1 forms part of a multi-protein complex, which mediates the DNA cleavage phase during V(D)J recombination, in the development of B- and T cells [24]. Knockout of Rag1 leads to the absence of mature B- and T cells [17]. The current literature does not attribute a direct functional role of Rag1 in macrophage polarization. In our study, we found that Rag1 KO mice showed an altered perivascular macrophage polarization towards more M1-like inflammatory macrophages. Similar data were obtained by Dywicki et al., where, in a mouse model of nonalcoholic steatohepatitis (NASH), Rag1 KO mice experienced a worse disease progression. This deterioration was accompanied by an increase in M1 phenotype inflammatory macrophages and a decrease in M2 phenotype anti-inflammatory macrophages [25]. Together, these results suggest that macrophage polarization in inflammatory animal models may be regulated by B- and T cells but not directly by Rag1.

B- and T cell cross-talk has been observed in mouse models of chronic inflammation. Additionally, B cells have been shown to possess immunosuppressive functions [26]. Despite being known as immunosuppressive cells, it was recently reported that the locally produced B cell metabolite, gamma-aminobutyric acid (GABA), can promote IL-10^+^ macrophages [27]. In light of this report, we hypothesize that the decreased arteriogenesis found in Rag1 KO mice may be caused by an altered macrophage niche. Rag1 KO mice in particular accumulated more M1-like macrophages in the perivascular region. This finding supports our theory that lymphocytes may promote M2-like macrophages in the perivascular region of developing collateral arteries.

The recovery of blood flow is important to prevent ischemic tissue damage. The arterioles branching out from the profunda femoris are the only source to restore blood flow in the lower leg. Therefore, it is necessary that arterioles expand to accommodate the required blood flow. Perivascular macrophages release growth-promoting factors and cytokines [5,22]. Accordingly, Rag1 KO mice exhibited reduced perfusion recovery, impaired vascular cell proliferation and a smaller luminal diameter. This observation supports our hypothesis that lymphocyte–macrophage coordination plays an important role in arteriogenesis.

Arteriogenesis plays a crucial role in preserving the function of vital organs [3]. Patients suffering from peripheral artery disease (PAD) face a higher risk of developing ischemic cardiovascular and limb complications. Furthermore, the risk of critical ischemic events is even higher in patients who underwent surgical management of lower extremity revascularization [28]. However, by creating natural bypasses, arteriogenesis limits adverse coronary scar formation and improves cardiac function [3]. Accordingly, arteriogenesis is a preferable alternative to the surgical management of arterial occlusive diseases. A study by Meier et al. found that coronary collateral artery growth reduced mortality by 36% in patients [29]. A clinical study on patients with myocardial infarction clearly demonstrated significant myocardial salvage when total coronary occlusion was bypassed by naturally grown collateral arteries [30]. Not all vascular disease patients are suitable for surgical management, therefore the therapeutic stimulation of arteriogenesis is an appealing means of treating those patients [31]. Given that macrophages are essential participants in the process of arteriogenesis, it is essential to comprehend the variables that influence macrophage activation, polarization, and functionality in order to develop non-invasive therapeutic approaches. Our current study found the essential role of Rag1 in macrophage regulation during arteriogenesis. Dissecting the role of immune cells in this process will enable a better understanding of the intricate mechanisms of arteriogenesis, facilitating the development of efficacious therapeutic agents to promote arteriogenesis in patients. Lymphocyte-targeted therapies have recently gained attention for reducing inflammation in occlusive diseases [11]. Our current study shows that the lack of functional lymphocytes in Rag1 KO mice impairs macrophage polarization and reduces arteriogenesis. Thus, it is important to note that lymphocyte-targeted approaches may also have an impact on the process of arteriogenesis.

Establishing the lymphocyte-specific niche in relation to perivascular macrophage polarization during arteriogenesis is of great importance. Although our study provides a systematic evaluation of the arteriogenic response in Rag1 KO mice, limitations remain in providing mechanistic insights. Therefore, further investigations are required to dissect the detailed mechanism of how lymphocytes influence the process of arteriogenesis and macrophage polarization. Moreover, understanding the precise roles of B- and T cells in this process is crucial. Together, the data obtained in the present study may provide the basis for future studies investigating in detail the function of individual subsets of lymphocytes in the process of arteriogenesis. 

## 4. Materials and Methods

### 4.1. Mice

C57BL/6 wild-type (WT) mice were purchased from Charles River Laboratories (Sulzfeld, Germany). Rag1 KO mice on a C57BL/6 background were bred and maintained at the animal facility of the Biomedical Center, LMU, Germany. Male mice, 8–12 weeks of age, were used for the experiments. All experiments were performed with the appropriate approval from the Bavarian Animal Care Use Committee (ROB-55.2Vet-2535. Vet_02_17-99). Mice were housed and fed under standard laboratory conditions and diet.

### 4.2. Femoral Artery Ligation and Laser Doppler Imaging

Arteriogenesis was induced by femoral artery ligation (FAL) according to the previously published protocol [23,32]. Briefly, after anesthetizing mice with a standard combination of fentanyl (0.05 mg/kg; CuraMED, Karlsruhe, Germany), midazolam (5.0 mg/kg; Ratiopharm, Ulm, Germany), and medetomidine (0.5 mg/kg; Pfister Pharma, Berlin, Germany), the skin of the upper thigh on both limbs was disinfected, and hair from the surgical site was shaved. Mice were placed on a heating pad (37 °C) and their limbs were fixed with surgical tape. The origin of the profunda femoris was estimated, and a small skin incision was made. The skin and subcutaneous layers were carefully separated to access the femoral artery from the femoral vein and nerve. A surgical thread was placed under the artery, distal to the epigastric and profunda femoris branch, and then securely tied. The same procedure was performed on the other limb (left leg) without closing the surgical thread, which served as an internal sham control. The skin incision was closed with a surgical suture. Laser Doppler imaging (Moor LDI 5061; Moor Instruments GmbH, Remagen, Germany) was used to analyze the perfusion of the hindlimbs. Imaging was conducted before the surgical procedure (baseline), immediately after femoral artery ligation (aFAL) to assess the accuracy of the ligation procedure. Imaging was performed after 3 days and 7 days aFAL to analyze the perfusion recovery. A mean flux value was generated from each limb from a defined region of interest (ROI). The perfusion recovery was calculated by dividing the occluded mean flux value by the sham-operated mean flux value, resulting in the occluded-to-sham operated ratio. A ratio value of 1.0 is equal to 100% perfusion. 

### 4.3. BrdU Treatment

Mice were injected i.p. with 100 µL of 5-Bromo-2′-deoxyuridine (BrdU) (1.25 mg; Sigma-Aldrich, St. Louis, MO, USA) dissolved in phosphate-buffered saline (PBS) starting after FAL and continuing daily until one day before the endpoint of the study. 

### 4.4. Perfusion and Tissue Harvesting

After laser Doppler imaging, mice were humanely sacrificed on day 3 and day 7 aFAL. Limbs were perfused with an adenosine buffer containing 1% adenosine, 5% bovine serum albumin (Sigma-Aldrich, St. Louis, MO, USA) dissolved in PBS, and 3% paraformaldehyde via the abdominal aorta as previously described [4]. Adductor muscles from the right and left limbs were excised and cryopreserved in TissueTeck O.C.T. (Sakura Finetek Germany GmbH, Umkirch, Germany) after overnight incubation in 30% sucrose.

### 4.5. Immunohistology

Cryopreserved muscle tissues were cut into 8–10 µm sections. For BrdU staining, a modified protocol was referenced [33]. Briefly, cryo-sections were first washed with PBS to remove excess TissueTek O.C.T. for 10–15 min at room temperature (RT). The sections were then permeabilized by incubating in 1N HCL at 37 °C for 30 min. After washing, the sections were subsequently incubated with anti-BrdU antibody (1:50, cat. ab6326; Abcam, Cambridge, UK) overnight in a humidified dark chamber at 4 °C. The following day, the sections were washed and incubated for 1 h at RT with the secondary antibody, goat anti-rat Alexa Fluor^®^546 antibody (1/200, cat. A21206; Invitrogen Thermo Fisher Scientific, Waltham, MA, USA). For lectin staining, the sections were incubated with lectin (1/200, cat. L4895; Sigma-Aldrich, St. Louis, MO, USA) for 30 min at RT. Lectins bind to glycoproteins of endothelial cell membranes and were used to identify arteries. To identify macrophages, the sections were incubated with anti-mannose receptor c-type 1 (MRC1) antibody (1/200, cat. ab64693; Abcam, Cambridge, UK) overnight at 4 °C, followed by secondary antibody staining with donkey anti-rabbit Alexa Fluor^®^546 antibody (1/200, cat. A10040; Thermo Fisher Scientific, Waltham, MA, USA). Further, the sections were incubated with an Alexa Fluor^®^488 anti-CD68 antibody (1/200, cat. ab201844; Abcam, Cambridge, UK) overnight at 4 °C. All sections were counterstained with DAPI (1/1000, cat. 62248; Thermo Fisher Scientific, Waltham, MA, USA) for 10 min at RT. The sections were then mounted with Dako fluorescent mounting medium (cat. S3203; Dako North America Inc., Carpinteria, CA, USA) and stored at 4 °C. Imaging was performed using a Leica epifluorescence microscope (DM6 B; Wetzlar, Germany). Ten superficial collateral images were acquired per mouse per group. The acquired images were analyzed using ImageJ (NIH, Bethesda, MD, USA). 

### 4.6. Statistical Analysis

Data were analyzed by using an unpaired Student’s *t*-test and a two-way ANOVA multiple comparison test using GraphPad Prism 8 (GraphPad Software, La Jolla, CA, USA). Results are expressed as mean ± standard error of the mean (S.E.M.). A *p*-value < 0.05 was considered as significant.

## Data Availability

The data are available from K.K. upon request.
